# Chromophore-Assisted Light Inactivation of Mitochondrial Electron Transport Chain Complex II in *Caenorhabditis elegans*

**DOI:** 10.1038/srep29695

**Published:** 2016-07-21

**Authors:** Andrew P. Wojtovich, Alicia Y. Wei, Teresa A. Sherman, Thomas H. Foster, Keith Nehrke

**Affiliations:** 1 University of Rochester Medical Center, Department of Anesthesiology, Rochester, 14642, United States of America; 2 University of Rochester Medical Center, Department of Pharmacology and Physiology, Rochester, 14642, United States of America; 3 University of Rochester Medical Center, Department of Medicine, Rochester, 14642, United States of America; 4 University of Rochester Medical Center, Department of Imaging Sciences, Rochester, 14642, United States of America

## Abstract

Mitochondria play critical roles in meeting cellular energy demand, in cell death, and in reactive oxygen species (ROS) and stress signaling. Most *Caenorhabditis elegans* loss-of-function (*lf*) mutants in nuclear-encoded components of the respiratory chain are non-viable, emphasizing the importance of respiratory function. Chromophore-Assisted Light Inactivation (CALI) using genetically-encoded photosensitizers provides an opportunity to determine how individual respiratory chain components contribute to physiology following acute *lf*. As proof-of-concept, we expressed the ‘singlet oxygen generator’ miniSOG as a fusion with the SDHC subunit of respiratory complex II, encoded by *mev-1* in *C. elegans,* using Mos1-mediated Single Copy Insertion. The resulting *mev-1::*miniSOG transgene complemented *mev-1* mutant phenotypes in *kn1* missense and *tm1081*(*lf*) deletion mutants. Complex II activity was inactivated by blue light in mitochondria from strains expressing active miniSOG fusions, but not those from inactive fusions. Moreover, light-inducible phenotypes *in vivo* demonstrated that complex II activity is important under conditions of high energy demand, and that specific cell types are uniquely susceptible to loss of complex II. In conclusion, miniSOG-mediated CALI is a novel genetic platform for acute inactivation of respiratory chain components. Spatio-temporally controlled ROS generation will expand our understanding of how the respiratory chain and mitochondrial ROS influence whole organism physiology.

Optogenetics uses light-sensitive proteins to induce a cellular response. Proteins such as channelrhodopsin and halorhodopsin conduct ions in response to light and have been used to control neuronal activity^1^. Another class of optogenetic tools is a group of proteins that generate reactive oxygen species (ROS) when illuminated^2^. These ROS-generating proteins, such as KillerRed^3^ and miniSOG^4^, are widely employed for their phototoxic effect. MiniSOG is a small flavoprotein derived from the LOV domain of Arabidopsis phototropin 2^4^. There are two variants of miniSOG, an active version that is able to produce ROS in response to light and an inactive variant that has a cysteine (Cys426) that prevents ROS generation^4^. MiniSOG has been used as a genetically-encoded photosensitizer for mutagenesis^5^, correlated light and electron microscopy^4^,^6^, cell ablation^7^,^8^ and inhibition of the synaptic proteins^9^. In general, expression levels and protein targeting greatly impact the function of genetic photosensitizers. For example, miniSOG’s ability to kill cells in response to light is highly dependent upon where the protein is targeted^7^.

Chromophore-Assisted Light Inactivation (CALI) is the selective inactivation of a target protein using a photosensitizer. Upon illumination the photosensitizer generates ROS, oxidizing susceptible residues and impairing protein function. The proximity of the photosensitizer to the target protein, its expression level, and illumination protocol contribute to the selectivity of CALI. Genetically-encoded photosensitizers such as miniSOG have the potential to allow for spatial and temporal control over protein inactivation by direct translational fusion with their coding regions. As with many conditional deletion approaches, CALI enables proteins whose expression is required for development or whose loss is lethal to be studied in the context of a live animal. An additional benefit is that CALI-mediated loss-of-function (*lf*) is acute; whereas conditional gene ablation or RNA interference’s effects depend upon turnover of existing gene products. The disadvantages are that *lf* can be incomplete, only mature proteins and not the gene or RNA are targeted, and light itself may have off-target effects. With these caveats in mind, we have developed a CALI approach to investigate the role of a mitochondrial respiratory chain component whose deletion is lethal.

Mitochondria generate the bulk of cellular energy through oxidative phosphorylation, whereby complexes of the electron transport chain generate a gradient that is used to produce ATP. Complex II (succinate:ubiquinone oxidoreductase) occupies a unique position within the mitochondrial metabolism machinery, being both a component of the electron transport chain and the Krebs cycle^10^. Complex II is comprised of four nuclear encoded subunits: SDHA-1, SDHB-1, SDHC-1 and SDHD-1^10^. SDHC-1, also known as MEV-1, is an integral membrane protein that anchors complex II to the inner membrane and helps coordinate the transfer of electrons onto coenzyme Q^10^. A point mutation in the *C. elegans* gene coding of SDHC-1, termed *mev-1*, increases ROS production and decreases lifespan and brood size^11^, while *mev-1*(*lf*) is lethal. These phenotypes have been recapitulated in an orthologous mouse model, suggesting evolutionary conservation^12^,^13^. Complex II dysfunction and ROS overproduction are implicated in Huntington’s disease^14^, cancer^15^, Charcot-Marie-Tooth disease type 2A^16^ and ischemic sensitivity^17^,^18^.

Here, we describe a *C. elegans* model where miniSOG has been fused to the complex II/MEV-1 subunit and integrated into the genome using single-copy insertion. The fusion transgene complements *mev-1* mutants, and CALI reduces complex II activity and succinate-driven respiration. Our results suggest that complex II is important for the organism’s responses to metabolic stress and for normal reproductive brood size, which may reflect metabolic potential. Our results also suggest that spermatozoa are selectively susceptible to reductions in complex II activity.

## Results

### Physiologic expression of miniSOG-tagged complex II subunit complements a mutant phenotype

**Figure 1:**
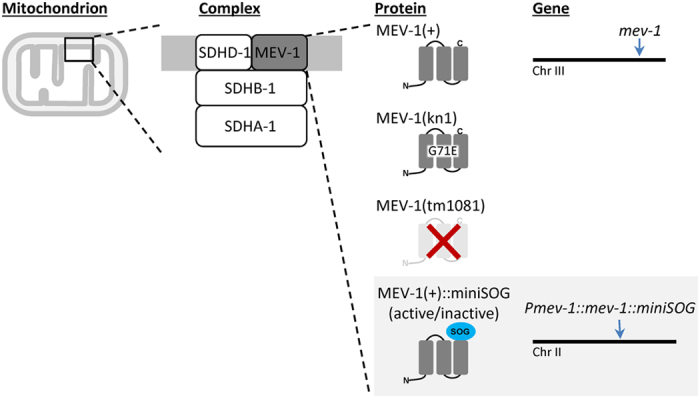
Schematic of how the genetically-encoded photosensitizer miniSOG is used for Chromophore-Assisted Light-Inactivation (CALI) of mitochondrial respiratory chain complex II. The *C. elegans* gene *mev-1* codes for one of four nuclear encoded subunits that comprise mitochondrial complex II, also known as mitochondrial succinate:ubiquinone oxidoreductase (in mammals, this complex is comprised of SDH subunits (A–D). The native *mev-1* gene (*mev-1*(+)) resides on chromosome III as indicated by the arrow. Two mutant alleles were used in this study, *mev-1*(*kn1*)*III* and *mev-1*(*tm1081*)*III*. The *kn1* allele contains a G71E point mutation resulting in a strain with a decreased brood size and sensitivity to paraquat^11^. The *tm1081* allele is a deletion that results in a lethal/sterile phenotype. The genetically-encoded photosensitizer miniSOG was inserted into a *mev-1* genomic fragment so as to create a fusion tag at the C-terminus, and the synthetic *mev-1*::minSOG transgene was integrated on chromosome II (arrow) using Mos1-mediated single-copy insertion (MosSCI). Either an active (able to produce ROS in response to light) or inactive (not able to produce ROS) variant of miniSOG was used to account for tagging and expression artifacts. Alleles are abbreviated as follows, *mev-1*(+)*III* as “+”, *mev-1*(*kn1*)*III* as “*kn1*”, *mev-1*(*tm1081*)*III* as “*tm1081*”, *rnySi29* as “*inactive*”, and *rnySi19* as “*active*”.

**Figure 2:**
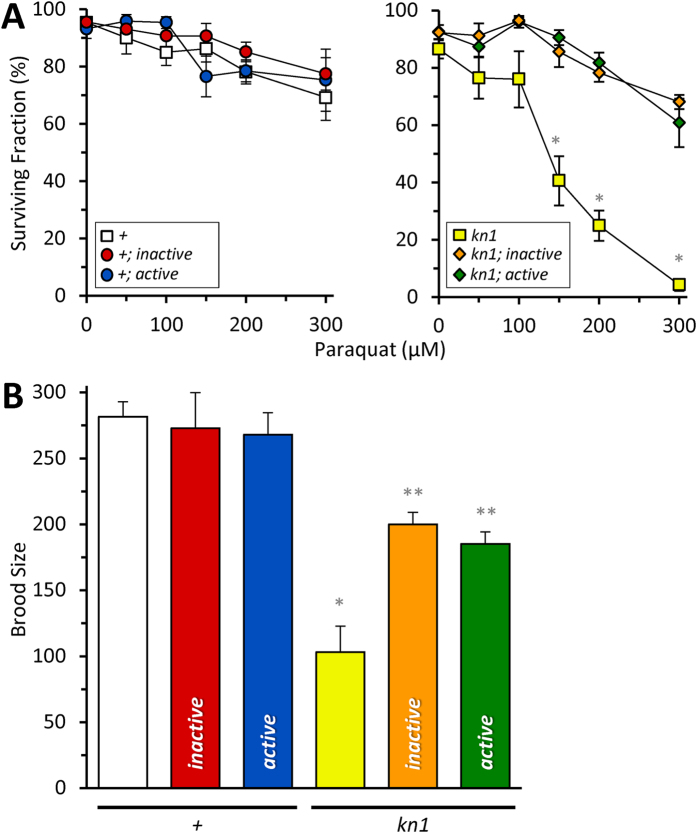
Complementation of *mev-1*(*kn1*)*III* mutant phenotypes with a transgenic *mev-1*(+)*::miniSOG* single copy insert. The *mev-1*::miniSOG fusion was expressed as a MosSCI single-copy transgene *in trans* to the wild-type or mutant *mev-1* on chromosome II, and strains that were homozygous for both *mev-1* loci were used to assess: (**A**) 96-hour survival as a function of cultivation on plates containing from 0–300 μM paraquat, as shown. Data from wildtype and *kn1* backgrounds are plotted on separate axes for clarity. Data are mean ± SEM. N = 4–22. An “N” is considered an independent trial of >50 worms. *Adjusted P < 0.01 vs all other groups (2-way ANOVA with Tukey multiple comparisons test). (**B**) Brood size, as represented by the number of viable progeny produced from staged L4 larval worms followed over the course of ten days. Data are mean ± SEM. N = 4–7. An “N” is considered an independent trial of up to 5 worms. *Adjusted P < 0.01 vs wild-type (+) and **Adjusted P < 0.05 vs wild-type (+) and *kn1* (1-way ANOVA with TUKEY multiple comparisons test). Alleles are abbreviated as outlined in Fig. 1.

SDHC-1, coded for by *mev-1* on chromosome III, is one of four nuclear-encoded complex II subunits. Mos1-mediated Single Copy Insertion (mosSCI) was used to express a *mev-1*::miniSOG fusion (including the *mev-1* promoter, genomic coding region and 3′ UTR) from the ttTi5605 Mos site in chromosome II, *in trans* to the native locus (Fig. 1). An inactive miniSOG variant^4^,^8^ was also expressed as a control. The integrated transgenes *rnySi29*, [*pTFA12*(*Pmev-1::mev-1::miniSOG*(*inactive*)*, unc-119 (C. briggsae*)) *into ttTi5605 ChrII Mos site]*, and *rnySi19,* [*pTFA11*(*Pmev-1::mev-1::miniSOG*(*active*)*, unc-119 (C. briggsae*)) *into ttTi5605 ChrII Mos site]* are hereafter referred to this as “*inactive*” and “*active*”, respectively. The virtue of using MosSCI is that the transgene fusion, being single copy, is expressed close to endogenous levels. We hypothesized that the MosSCI approach would allow us to avoid overexpression artifacts, including stoichiometric disequilibrium between subunits, and would limit the effects of miniSOG-generated ROS to disrupting complex II function. In fact, both the active and inactive fusions were well-tolerated, and neither fusion impaired development or reproduction in a wild-type *mev-1* background (Fig. 2A), suggesting the absence of a dominant negative effect.

To test whether the *mev-1*::miniSOG fusions were active, we assessed their ability to complement a *mev-1* mutant. The *mev-1*(*kn1*) allele contains a point mutation that results in an increased complex II ROS production, decreased brood size and increased sensitivity to paraquat^19^. We crossed the miniSOG fusions into the mutant background and found that both *mev-1*::miniSOG(*active*) and *mev-1*::miniSOG(*inactive*) variants rescued the *kn1* paraquat sensitivity (Fig. 2A). Moreover, *kn1* brood size was also restored by both fusions, albeit below wild-type levels (Fig. 2B and Supplementary Figure S1). Hence, the *active* and *inactive* miniSOG variants were always assessed in parallel to control for light-independent effects.

**Figure 3:**
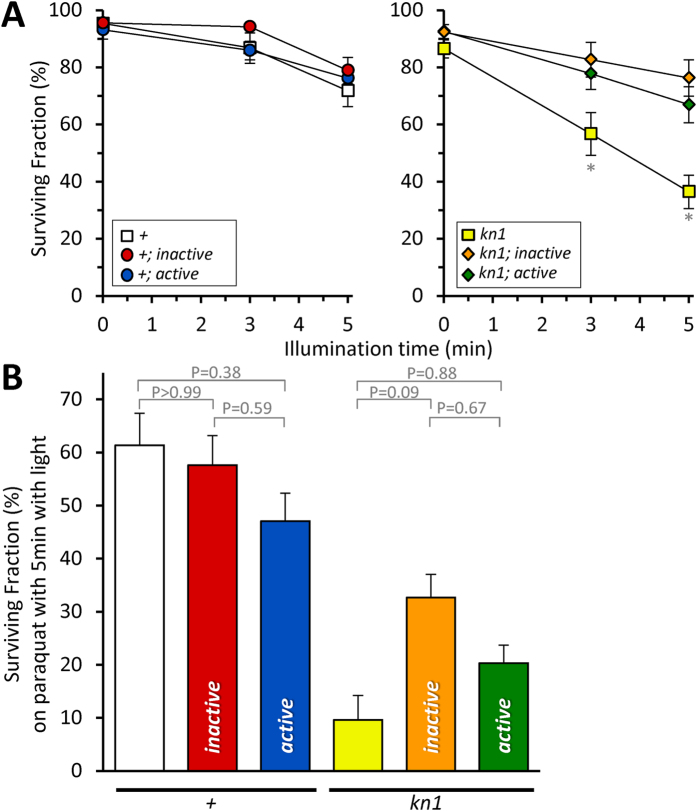
Light-induced phenotypes in a *mev-1*(*kn1*)*III* mutant background. The same strains as shown in Fig. 2 were used to assess: (**A**) 96-hour survival as a function of light. Staged L1 larvae were illuminated for the indicated amount of time, and survival was scored four days later. *Adjusted P < 0.05 vs all other groups at that light treatment (2-way ANOVA with TUKEY multiple comparisons test). Data from wildtype and *kn1* backgrounds are plotted on separate axes for clarity. Data are mean ± SEM. N = 8–18. An “N” is considered an independent trial of 25–100 worms. (**B**) 96-hour survival as a function of cultivation on plates containing from 200 μM paraquat. Adjusted P values are indicated (one-way ANOVA with Tukey multiple comparisons test). Data are mean ± SEM. N = 8–18. An “N” is considered an independent trial of 25–100 worms. Alleles are abbreviated as outlined in Fig. 1.

*C. elegans* depend on mitochondrial function for development. We hypothesized that CALI would selectively influence development in *kn1*; *active* worms, but not in *kn1*; *inactive* worms. Surprisingly, blue light (470 ± 20 nm, 10.2 mW/mm^2^) caused modest but significant dose-dependent reduction in L1 survival in all strains, including the wild-type control (Fig. 3A, adjusted P < 0.05, 2-way ANOVA, Tukey), but there were no differences between the wild-type (+) and miniSOG expressing strains. The *kn1* mutant on its own was particularly sensitive to blue light and 5′ application resulted in ~60% reduction in survival, which was significantly different from all other strains. Both the *inactive* and *active* miniSOG variants complemented this *kn1* phenotype (Fig. 3A). However, as observed in the wild-type background, there was no difference between the variants with light exposure. This result highlights the potential toxicity of blue light, which has been observed particularly with respect to mitochondrial function previously^20^, and suggests that the *kn1* mutant sensitizes worms to its toxic effects.

The observation that CALI did not phenocopy the *kn1* mutant suggested several possibilities. First, photo-inactivation of the fusion construct may be incomplete. As per heteroplasmic load tolerance with respect to mitochondrial DNA mutants^21^, it is possible that partial loss of the rescue fusion nevertheless provided enough “wild-type” protein to suppress the *kn1* mutant phenotype.

Moreover, larval development may not be particularly susceptible to acute reductions in complex II activity. However, we noted that although there was not a significant difference between them, the *kn1*; *active* worms trended towards reduced survival in response to light compared to the *kn1*; *inactive* worms (Fig. 3A). Given these caveats, we reasoned that a CALI phenotype might be revealed by subjecting the worms to an additional stress whose effects are known to be exacerbated by the *kn1* mutant. The *mev-1*(*kn1*) allele was originally isolated in a genetic screen for mutants that were susceptible to methyl viologen, or paraquat^19^. Consistent with this reasoning, in the presence of paraquat, CALI of *kn1*; *active* reduced survival to a point indistinguishable from the *kn1* mutant alone (Fig. 3B). However, CALI also reduced survival in *kn1*; *inactive*, though not to a similar extent (Fig. 3B). With the background effect of both the fusion transgenes and blue light toxicity, as well as the possibly confounding gain-of-function effects of *kn1* itself, we decided to simplify our model by expressing the *mev-1*::miniSOG fusions in a *mev-1*(*lf*) background.

**Figure 4:**
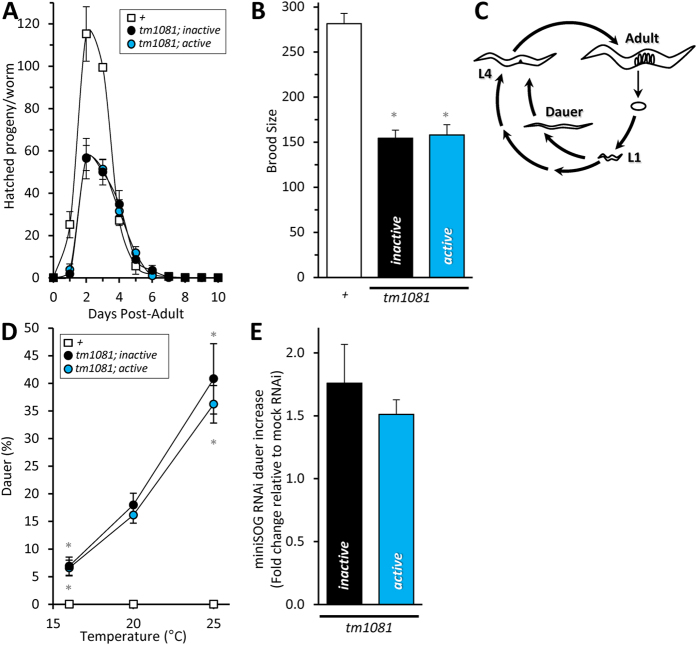
Complementation of a *mev-1*(*tm1081*)*III* deletion mutant with a transgenic *mev-1*(+)*::miniSOG* single-copy insert. The loss of *mev-1* is lethal, hence *tm1081* can only be maintained as a heterozygote through the use of a genetic balancer. Here, the *mev-1*(+)::miniSOG fusion was expressed *in trans* to the *tm1081* deletion on chromosome II, and worms that were homozygous at both loci were used to assess: (**A**) Daily offspring production from staged L4 larvae and (**B**) The total number of viable progeny per worm. Data are mean ± SEM. N = 4–7. An “N” is considered an independent trial of up to 5 worms. *Adjusted P < 0.05 vs N2 (1-way ANOVA with TUKEY multiple comparisons test). Wild-type (+) brood size is reproduced from Fig. 1B for comparison. (**C**) A schematic representing the *C. elegans* life cycle. *C. elegans* progress through four larval stages (with intervening molts) to become a reproductively mature adult. Following the L1 larval stage, worms can arrest in an alternative “dauer” stage, which generally occurs in response to limiting factors in their environment such as a lack of food or crowding. Dauer larva are highly resistant to stress, age very slowly, and have an altered metabolism. (**D**) Temperature-dependent dauer entry in *tm1081* mutants complemented using *mev-1*::miniSOG transgenes. Worms were synchronized via a 2-hour egg lay. Eggs were seeded onto a NGM plated with OP50 and incubated for the indicted time. Two days post-egg lay, the number of dauer-arrested worms was scored. Data are mean ± SEM. N = 6–9. *Adjusted P < 0.05 vs 20 °C per genotype (2-way ANOVA with Tukey multiple comparisons test). (**E**) RNAi targeting of miniSOG exacerbates the dauer phenotype. Data are mean ± SEM. N = 3. The second generation grown on RNAi for miniSOG was lethal. Alleles are abbreviated as outlined in Fig. 1.

As expected, both the *mev-1*::miniSOG *active* and *inactive* fusions complemented lethality in a *mev-1*(*tm1081*) deletion mutant (Fig. 4). To confirm this result, RNAi against miniSOG was used to suppress complementation. The first generation on RNAi plates exhibited increased dauer formation (Fig. 4E; *tm1081*; *inactive*, 76 ± 31% increase over scrambled; *tm1081*; *active*, 51 ± 12% increase over scrambled), a *C. elegans* developmental phenotype that occurs in response to harsh environmental conditions such as population density, food supply and temperature^22^. By the second generation, miniSOG RNAi phenocopied the *tm1081* mutant and caused overt death. No effect was seen in the wild-type (+) strain. Interestingly, mitochondrial dysfunction can be interpreted as a xenobiotic or pathogen attack^23^, and we noted that the *tm1081*; *inactive* and *tm1081*; *active* strains both avoided the miniSOG expressing RNAi bacterial lawn.

All of the rescued mutants grew to reproductive adulthood in roughly the same period as wild-type worms. However, both the *tm1081*; *inactive* and *tm1081; active* worms had a delayed egg laying period and decreased brood size (Fig. 4A,B). Of the eggs that were laid, 81 ± 2% (*tm1081*; *inactive*) and 81 ± 3% (*tm1081*; *active*) hatched. Moreover, we observed an increased propensity to undergo dauer arrest in both the *tm1081*; *active* and *tm1081*; *inactive* worms despite being uncrowded and well-fed (Fig. 4C). Larva can remain in dauer for up to four months^24^; however, the dauer arrest was transient (Supplementary Figure S2) with all the worms exiting dauer by 11 days. Hence, consistent with our previous observations using the *mev-1*(*kn1*) allele (Fig. 2), the complemented worms exhibited mild abnormalities consistent with incomplete rescue.

### Chromophore-assisted light inactivation (CALI) of complex II activity

**Figure 5:**
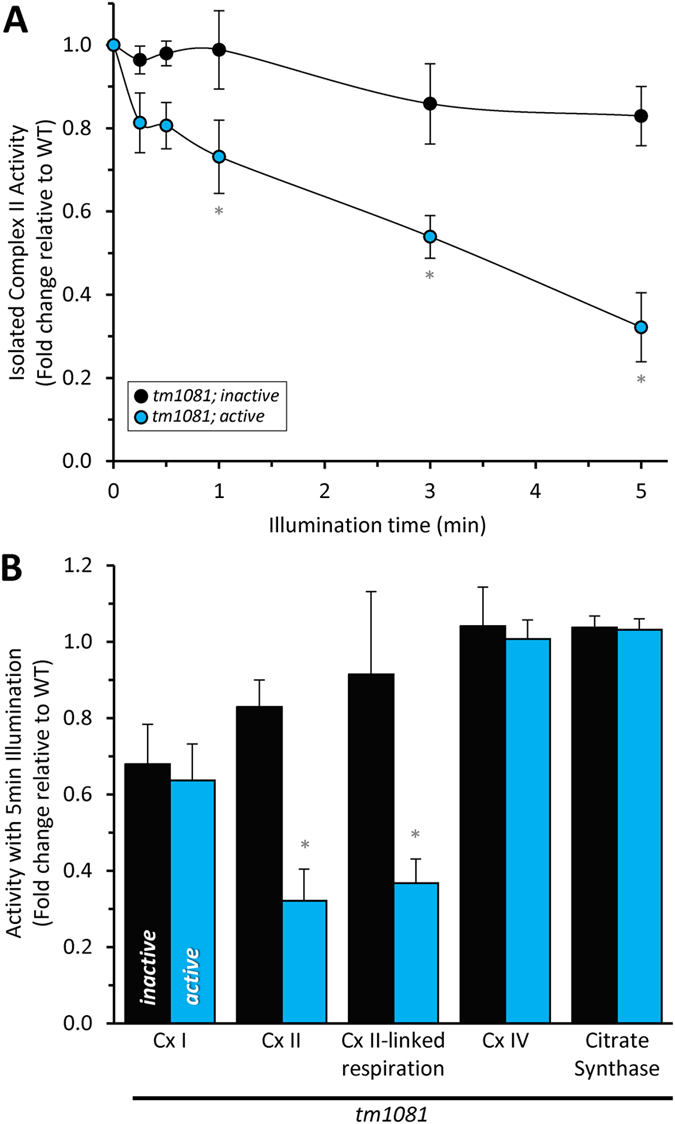
Light-induced loss of complex II activity. Mitochondria were isolated from wild-type (+), *tm1081*; *inactive*, and *tm1081*; *active* strains. The isolated mitochondria were then exposed to varying periods of illumination and: (**A**) Assayed for complex II (succinate-ubiquinone reductase) activity. Data at all time points were normalized to wild-type (+) values, to account for miniSOG-independent effects of blue light, as well as to baseline complex II activity for each strain. The “pre-light” values (in nmol DCPIP/min/mg) were: wild-type (+), 13.5 ± 2.6; *tm1081*; *inactive*, 9.2 ± 0.5; and *tm1081*; *inactive*, 10.9 ± 0.9. *Adjusted P < 0.05 vs the no light or vs the light-matched inactive counterpart (2-way ANOVA with Sidak multiple comparisons test). N = 5–7. (**B**) Assayed for the activity of other mitochondrial enzymes, complex I (NADH:ubiquinone oxidoreductase), complex IV (cytochrome c oxidase) and citrate synthase. Exposure to light was for 5 min and the data was normalized as in panel A. Baseline complex I activity (N = 5–7): wild-type (+), 77 ± 2.4 nmol NADH/min/mg; *tm1081*; *inactive*, 131 ± 14; *tm1081*; *active*, 121 ± 9. Baseline complex II-linked respiration (n = 9–10): wild-type (+), 29.5 ± 2.8 nmol O_2_/min/mg; *tm1081*; *inactive*, 12.1 ± 1.0; *tm1081*; *active*, 12.6 ± 1.0. Baseline complex IV activity (N = 7–10): wild-type (+), 35.4 ± 3.1 nmol O_2_/min/mg; *tm1081*; *inactive*, 34.3 ± 2.3; *tm1081*; *active*, 34.3 ± 1.6. Baseline citrate synthase activity (N = 5–18): wild-type (+), 356 ± 19 nmol DTNB/min/mg; *tm1081*; *inactive*, 568 ± 31; *tm1081*; *active*, 538 ± 31. *Adjusted P < 0.01 vs light-matched inactive counterpart (2-way ANOVA with Sidak multiple comparisons test). All data are mean ± SEM. An “N” is an independent mitochondrial isolation. Alleles are abbreviated as outlined in Fig. 1.

Directly assessing CALI of complex II required that we account for the light-independent effects of the *mev-1*::miniSOG transgenes as well as the transgene-independent basal effects of blue light itself. Hence, the remaining data has been normalized first to the illuminated wild-type background to account for blue light and second to the unilluminated *tm1081*; *inactive* strain to account for the transgene. A titratable light-dependent inhibition of complex II activity was observed in mitochondria isolated from *tm1081*; *active* but not *tm1081*; *inactive* or wild-type worms (Fig. 5). This inhibition occurred in both an isolated complex II activity (Fig. 5A) and in succinate-driven respiration (Fig. 5B). Despite inhibition failing to reach 100%, exposure to blue light beyond 5 min resulted in non-specific effects that overshadowed our ability to assess acute complex II inactivation by CALI.

The specificity of complex II inactivation was investigated by measuring the activity of neighboring proteins, including complex I (NADH:ubiquinone oxidoreductase), complex IV (cytochrome c oxidase) and citrate synthase (Fig. 5B). Following 5 min blue light exposure, there were no differences between the *tm1081*; *inactive* and *tm1081*; *active* strains. We conclude that the extent of miniSOG-mediated CALI was restricted to complex II, as would be expected given its high reactivity and limited radius of action.

Since the loss of *mev-1* is lethal we hypothesized that CALI in live worms would, like miniSOG RNAi, phenocopy the *tm1081* mutant and cause them to die. However, we observed no lethality in L1 or adult *C. elegans* subject to acute light exposure (Supplementary Figure S3). *De novo* synthesis of *mev-1::miniSOG* or mitogenesis may overcome lost complex II activity, hence suppressing a more overt phenotype. We tested this hypothesis by illuminating L1 larva (a non-lethal insult) and growing them on miniSOG RNAi plates (non-lethal in the first generation) to prevent reconstitution of lost MEV-1 through de novo synthesis. We found no significant difference between the groups (Supplemental Figure S4). However, it is possible that the relevant *mev-1* target is in neurons, which are refractory to RNAi. Therefore, we also investigated the cumulative effect of consecutive illumination in adult *C. elegans.* Three pulses of light provided at 2 hour intervals elicited similarly unremarkable effects as a single pulse (Supplemental Figure S5). However, three pulse provided at 24 hour intervals resulted in a subtle developmental phenotype, with fewer L1 progressing to adulthood (Supplemental Figure S6). This latter result supports the idea that mitogenesis may over time help to suppress the effect of *mev-1* CALI.

**Figure 6:**
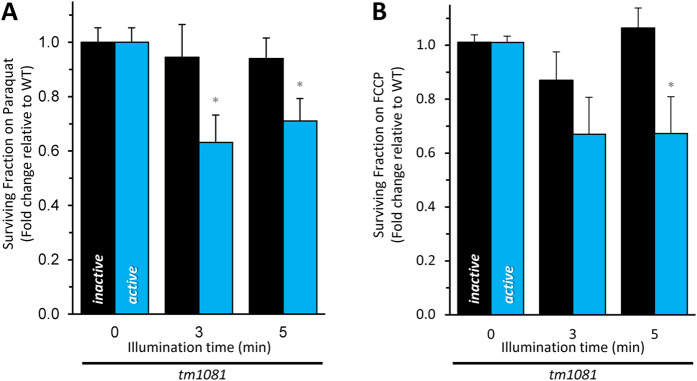
CALI of complex II sensitizes *C. elegans* to mild stress. Wild-type (+), *tm1081*; *inactive*, and *tm1081*; *active* strains were: (**A**) illuminated as L1 larvae for the indicated period and cultured on NGM plates containing 200 μM paraquat. 96-hour survival is plotted relative to the zero-illumination baseline. Baseline survival on 200 μM paraquat was wild-type (+), 78 ± 4%; *tm1081*; *inactive* , 68 ± 4%; *tm1081*; *active*, 60 ± 3%. Data are mean ± SEM. N = 11–13. An “N” is considered an independent trial of >50 worms. *Adjusted P < 0.05 vs the no light or vs the light-matched inactive counterpart (2-way ANOVA with Sidak multiple comparisons test). (**B**) Synchronized L4 were illuminated for the indicated time and cultured on NGM plates seeded with OP50 containing 1 μM FCCP. Viability was assessed 1 day later and plotted relative to zero-illumination baseline. Baseline survival on 1 μM FCCP was wild-type (+), 99 ± 1% survival; *tm1081*; *inactive*, 93 ± 3%; *tm1081*; *active*, 94 ± 2%. Data are mean ± SEM. N = 9. An “N” is considered an independent trial of >50 worms. *Adjusted P < 0.05 vs the no light or vs the light-matched inactive counterpart (2-way ANOVA with Sidak multiple comparisons test). Data were normalized to wild-type (+) to account for miniSOG-independent effects of blue light. Alleles are abbreviated as outlined in Fig. 1.

In addition to mitogenesis, if we postulate that the reduction in complex II activity observed in mitochondria (Fig. 5) reflects activity in the intact worm, the complex II activity remaining after CALI may suffice to support viability. It is also possible that other metabolic pathways can compensate for the loss of complex II activity. Interestingly, mimicking a metabolic stress with either paraquat or the proton ionophore FCCP to partially uncouple mitochondria unveiled a cryptic light-dependent effect on survival in the *tm1081*; *active* strain (Fig. 6 and Supplementary Figure S7). These data suggest that in the short term complex II is necessary to meet increased metabolic demands.

### CALI of complex II results in a decreased brood size

**Figure 7:**
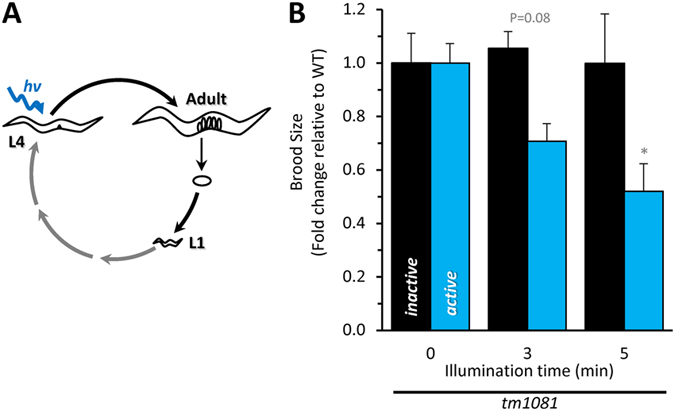
CALI of complex II decreases brood size. (**A**) Schematic of *C. elegans* life cycle and light exposure. Staged L4 worms were illuminated (day 0) for the indicated time. (**B**) Brood size is represented by the total number of viable progeny produced from staged, illuminated L4 larval worms followed over the course of ten days, expressed as a fold change relative to wild-type (“+”) to account for miniSOG-independent effects of blue light and plotted relative to the zero-illumination baseline brood size for each independent strain. Baseline values were 282 ± 11 progeny for “+”, 143 ± 16 progeny for *tm1081; inactive* and 151 ± 11 progeny for *tm1081; active*. Daily progeny counts are presented in Figure S8. Data are mean ± SEM. N = 4–6. An “N” is considered an independent trials of 1–5 worms per trial. *Adjusted P < 0.01 vs light-matched inactive counterpart 2-way ANOVA with Sidak multiple comparisons test). Alleles are abbreviated as outlined in Fig. 1.

The *mev-1*(*kn1*) mutant has a reduced brood size (Fig. 2B), which is complemented only partially by the *mev-1*::miniSOG transgenes (Fig. 4B), suggesting a role for complex II in the production of offspring. A single worm produces ~300 embryos, and there is intense metabolic demand during the egg-laying period; approximately the entire body mass of the worm is converted to oocytes in a 24 hour period. Embryo production is supported by mitogenesis in both somatic and germ cells late during larval maturation^25^. We illuminated staged late L4 larva with blue light (Fig. 7A) and scored the number of viable progeny over a ten day period (Supplementary Figure S8). Light caused a dose-dependent decrease in the number of *tm1081*; *active* progeny relative to the *tm1081*; *inactive* and wild-type controls (Fig. 7B).

Since this effect could be attributed to either the loss of somatic or germ cell complex II activity, we tested if mating could suppress the light-induced loss of brood size. The hermaphrodite worm is sperm-limited. Mating can increase the number of progeny, but more importantly in this case, if the reduction in brood size was caused by defective sperm mitochondria, we hypothesized that cross fertilization would suppress this phenotype.

**Figure 8:**
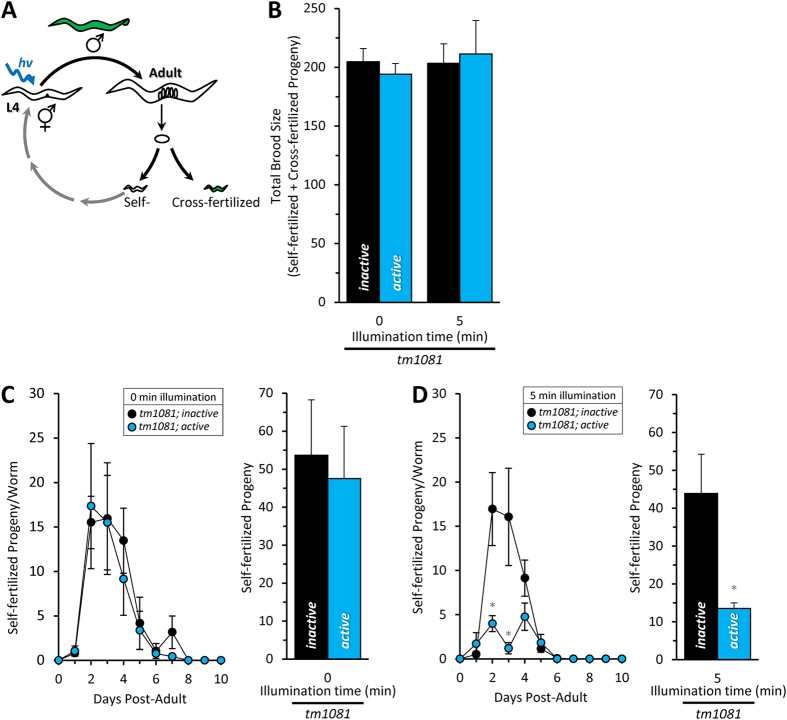
Mating restores CALI-induced decrease in brood size. (**A**) Schematic of *C. elegans* life cycle, light exposure and mating. Staged L4 worms were illuminated (day 0) for the indicated time and placed individually onto NGM plates seeded with minimal OP50 (i.e. a mating plate), with males containing an integrated fluorescent transgene. Worms were moved daily to fresh plates over the course of 10 days. Each plate was then scored for self-fertilized (non-fluorescent) and cross-fertilized (fluorescent) progeny. (**B**) The total brood size is the sum of self-fertilized and cross-fertilized progeny over the 10 day period. Mating did not cause a significant increase in the total brood size in the absence of light (unmated values presented in Fig. 4B; adjusted P > 0.05, Tukey multiple comparisons test), suggesting that sperm are not the limiting factor in the light-independent brood size reduction. (**C**) The number of self-fertilized progeny in mated *tm1081; inactive* was no different than in *tm1081; active* worms in the absence of light (p = 0.76 unpaired 2 tailed t-test), suggesting that the sperm in these two strains are equivalently functional at baseline. (**D**) However, following 5-min exposure to blue light there was a significant decrease in the self-progeny at day 2 and 3 (adjusted P < 0.0001 vs day-matched inactive counterpart, 2-way ANOVA with Sidak multiple comparisons test) and total self-fertilized progeny (*P = 0.02 unpaired 2-tailed t test), suggesting that sperm are particularly susceptible to complex II CALI in this model. All data are mean ± SEM. N = 6–8. An “N” is considered an independent trials of 1–5 worms per trial. Alleles are abbreviated as outlined in Fig. 1.

Males carrying an integrated GFP reporter (P*dat-1*::GFP) were used to distinguish cross-fertilized progeny from self-fertilization (Fig. 8A). Mating resulted in a significant increase in the brood size of wild-type worms (mated *wild-type*, 493 ± 78 total progeny vs unmated *wild-type* unmated 282 ± 11 total progeny as shown in Fig. 2A, adjusted P < 0.01, 2-way ANOVA with Tukey multiple comparisons test). However, there was no significant increase in either the *tm1081*; *inactive* or *tm1081*; *active* total brood sizes with mating (Fig. 8B, unmated data in Fig. 4B, adjusted P = 0.70 and 0.76, respectively 2-way ANOVA with Tukey multiple comparisons test), suggesting that the number of oocytes that can be successfully fertilized is limited in these strains. As oogenesis itself is an energy demanding process, we hypothesize that this is due to an underlying light-independent metabolic defect in the transgenic strains, possibly reflecting incomplete complementation of the null mutant.

While both male and hermaphrodites have sperm, male-derived spermatozoa are larger and outcompete hermaphrodite-derived spermatozoa^26^. In this regard, the wild-type hermaphrodites had an almost full suppression of self-fertilization resulting in 91 ± 4% cross progeny. For unknown reasons, this value was reduced to 71 ± 9% and 73 ± 3% in the *tm1081*; *inactive* and *tm1081*; *active* hermaphrodites, respectively. Due to this disparity, the data were not normalized to wild-type, and hence the miniSOG-independent effects of blue light are not accounted for. However, even so, it is clear that light selectively decreased the number of self-fertilized progeny in the *tm1081*; *active* strain but not the *tm1081*; *inactive* strain (Fig. 8C,D). More importantly, this effect was specific to self-fertilized progeny, as cross-progeny brood size exhibited no light-dependence in the *tm1081*; *active* strain (Fig. 8B). These results suggest that CALI of spermatozoa complex II and loss of mitochondrial function in these specific cells contributed to the miniSOG-mediated effects of blue light on brood size.

## Discussion

miniSOG is a versatile optogenetic tool that has been used for electron microscopy^4^,^6^, cell ablation^7^,^8^, mutagenesis^5^ and CALI of synaptic proteins^9^. The precise mechanism of miniSOG photosenstization remains unclear, as reports on the type of ROS and quantum yield vary^2^,^4^,^7^,^27^,^28^. Moreover, the prevailing photosensitization mechanism *in vitro* may differ under *in vivo* conditions and the mechanism could be the result of a combination of photosensitization processes. MiniSOG was first described as a singlet oxygen generator^4^. This type of ROS is highly reactive and does not directly convert with endogenous ROS species such as superoxide or hydrogen peroxide. To develop a system for the selective inactivation of complex II of the mitochondrial respiratory chain, we used miniSOG for CALI of the SDHC subunit, coded for in worms by *mev-1*. A simple transgene array containing multiple copies of the *mev-1*::miniSOG fusions was deemed likely to generate overexpression artifacts. Hence, we incorporated our transgene into the ttTi5605 loci on chromosome II using MosSCI for single copy expression.

The miniSOG-tagged *mev-1* fusions were functional and could complement two independent *mev-1* mutant alleles, both the *kn1* missense mutant which generates high levels of superoxide, as well as the *tm1081* deletion mutant. Although miniSOG is comparatively smaller than other photosensitizers^4^, the tag may nevertheless impede the organization or function of complex II. This is consistent with the incomplete phenotypic suppression observed in brood size with both the active and inactive miniSOG transgenes, independent of light (Figs 2 and 4). In addition, the *C. elegans* life cycle has checkpoints that are influenced by environmental conditions such as food availability^29^. Nutritional scarcity during early development can result in growth arrest or entry into the dauer stage. During the L1 to L2 transition worms shift reliance from the glyoxylate cycle (complex II independent) to aerobic metabolism (complex II dependent)^30^. Dauer worms do not exhibit this metabolic shift. The appearance of a transient dauer phenotype in the *tm1081*; *inactive* and *tm1081*; *active* strains may be the result of decreased expression/activity of complex II resulting in a perceived food scarcity. Hence, our ability to normalize for light-independent effects of transgene rescue using the inactive *mev-1*::miniSOG fusion strain was a critical control.

We also found that blue light exposure itself results in adverse consequences on development and fertility. miniSOG requires a flavin mononucleotide cofactor, and flavins are critical to mitochondrial biology^4^. We hypothesize that blue light can negatively impact the flavin-rich mitochondria, though the specific consequences on individual components of mitochondrial function are as-of-yet unknown. We note that there is a ~35% reduction in complex I activity in response to illumination in both the *tm1081*; *inactive* and *tm1081*; *active* strains (Fig. 5B). *C. elegans* also have neurons that mediate behavioral responses to blue light^31^. However, it is less likely that these neurons are the physiologic basis of the blue light sensitivity, as the effect is manifest in purified mitochondria as well as the whole organism. Longer periods of illumination in our hands elicited confounding effects, and short bursts of intense blue light may be optimal for CALI approaches. Recently, mutations to miniSOG have rendered a version that *in vitro* has a larger quantum efficiency of singlet oxygen generation^32^. This advance may decrease the blue light exposure time as well as the potential for nonspecific effects. In addition, genetically-encoded photosensitizers that respond to longer wavelength light may elicit fewer potential off-target effects^3^,^33^.

While genetic ablation of *mev-1* is lethal, the acute loss of complex II by CALI is survivable. We acknowledge that CALI-mediated complex II loss-of-function is likely not complete (Fig. 5), while metabolic alternatives may also compensate in the short-term and mitogenesis in the long term. *C. elegans* have been shown to exhibit remarkable metabolic plasticity and adapt astoundingly to their environment. For example, alternative energy pathways such as the glyoxylate cycle^30^ may shift metabolism away from the electron transport chain and ultimately decrease complex II dependence^30^. A strain containing *gas-1*(*fc21*) has a reduction-of-function mutation in a complex I subunit that is accompanied by a compensatory upregulation of complex II^34^,^35^, suggesting that some level of crosstalk occurs between these two conduits to the electron transport chain. It is also notable that attempts to obtain a *gas-1*(*fc21*) double mutant with either *tm1081*; *inactive* and *tm108*; *active* were unsuccessful. In the long term, respiratory chain damage could induce compensatory responses. Many of the canonical *mit* mutants in *C. elegans* exhibit constitutive activation of the mitochondrial unfolded protein response and require this adaptive response to remain viable^36^. While we do not observe induction of a mitochondrial chaperone *hsp-60*::GFP reporter following complex II CALI, we cannot rule out an age-dependent compensatory effect through this or other pathways.

Our data in general showed that CALI of complex II rendered worms sensitive to mild metabolic stresses. It is likely that the loss of complex II resulted in worms that are unable to respond to increased metabolic demand. It is unclear, however, whether this applies selectively to complex II or whether the same holds true for other bioenergetic measures. CALI of complex II also selectively impairs spermatozoa, resulting in reduced brood size. Spermatogenesis occurs both in hermaphrodites and males and begins at early L4 larval stage^37^. During development, hermaphrodites switch from spermatogenesis to oogenesis and are therefore sperm limited. Hence, the introduction of male sperm can increase the number of progeny. During self-fertilization, many spermatozoa are pushed from the spermatheca to the uterus, requiring the spermatozoa to crawl back^37^. Spermatozoa contain about 50 to 70 mitochondria^38^, and may be considered translationally silent^39^. We hypothesize that complex II is important in meeting the energy demand necessary for motility and spermatozoa are unable to cope with the loss of complex II. In fact, many nuclear-encoded respiratory genes exhibit a strong maternal effect loss of function phenotype, and RNAi of these gene products exerts a more potent effect than is observed in null animals born to heterozygous parents^40^,^41^. We hypothesize further that because of the limited period over which fertilization and embryonic development occurs, acute inactivation of respiratory function is more likely to have pronounced effects in germ cells, where there is less time to adapt.

Overall, we have demonstrated an approach to selectively inactivate a mitochondrial respiratory complex with light. This model facilitates studying the contribution of individual respiratory chain components whose *lf* phenotypes have heretofore been limited to RNAi^42^,^43^,^44^. The development of more efficient photosensitizers with different spectral responses may increase the utility of this technique, and perhaps allow us to distinguish *lf* effects from the effects of ROS itself.

## Methods

### *C. elegans* Strains, Alleles and Culturing Techniques

*C. elegans* were cultivated using standard laboratory practices^45^. Unless indicated otherwise, all strains were grown at 20 °C on Nematode Growth Media (NGM)-agar plates seeded with the bacterial strain OP50 as a food source. In addition, strains were grown in the dark (though routine propagation utilized transmitted light stereomicroscopes to move worms). N2 (Bristol) was used as the wild-type strain. A complete list of mutants and strains used in this work is available in Supplementary Table S1. Strain abbreviations used throughout the text are summarized in Fig. 1 and the Supplementary Table S1.

The *mev-1*::miniSOG transgenesis was accomplished through Mos-mediated single-copy insertion^46^. Briefly, the coding region of miniSOG (courtesy of Drs. Roger Tsien and Yishi Jin, University of California, San Diego) was inserted between the *mev-1* promoter/genomic coding region and the translational stop site/3′ UTR to code for a fusion protein where SDHC is tagged with active miniSOG (pTFA9 vector) or inactive miniSOG (pTFA10 vector) at its C-terminus. An *Spe*I fragment from these vectors was inserted into the MosSCI base vector pCFJ151^46^ to create pTFA11 and pTFA12, which were injected into the gonads of *unc-119* mutant worms and integrated at the ttTi5605 Mos locus on chromosome II using positive and negative selection as described previously^46^. Following extensive outcrossing, the genomic loci at the insertion sites were PCR amplified and sequenced fully to verify the absence of indels or mutations.

The vector for miniSOG RNAi contains from ~35–280 of the miniSOG coding region, cloned as a PCR-tagged *BamH*I-*Xba*I fragment into the base vector pPD129.36 (courtesy of A. Fire).

Conventional mating protocols were used to cross the MosSCI transgenes (chromosome II) with *mev-1* mutants (chromosome III). Genotyping was through single worm PCR. The *kn1* allele is a point mutation in which a G becomes an A and introduces a genomic *BspE*I site. mev-1 geno F1 (TTT TTG TGT ACG CAT GAA GGA GAA) and mev-1 geno R1 (AGG GCT GGG GCG GAG TAA GAA) primers were used to PCR amplify a 632 nt fragment. Digestion with *BspE*I results in a 194nt and a 438nt fragment in the *kn1* mutant. For the deletion allele *tm1081* a combination of three primers was used, mev-1 geno F1, mev-1 geno F3 (TGG CTG TCC ATT TGG AAG CAC CC) and mev-1 geno R1. The expected product size is 1172 nt and 632 nt from wildtype and 300 nt for the *tm1081* deletion. The MosSCI transgene was amplified using OG967 and OG970 primers flanking the ttTi5605 recombination interval, as described^46^. Repeat genotyping of multiple offspring in later generations was used to confirm homozygosity.

### RNA interference (RNAi)

RNAi was performed as previously described^47^. Briefly, HT115 bacteria were transformed with either an empty vector or vector targeting miniSOG, as above. The bacteria were grown at 37 °C to midlog phase, induced with 1 mM isopropyl-β-D-thiogalactosidase (IPTG) for 1 h, and seeded on NGM plates that also contained 1 mM IPTG. Three days later, L3 larval worms (F0) were placed on the RNAi plates, moved to new plates at 24 h, and their progeny (F1) were screened for phenotypes. The F1 generation was then moved to a fresh plate, and their progeny (F2) were also screened.

### Illumination

We employed an Olympus MVX stereomicroscope to expose worms to blue light using X-Cite^®^ (Excelitas Technologies Corp., Waltham, MA) illumination filtered through a GFP filter set (470-nm excitation ± 20-nm; Chroma, Bellows Falls, VT). Worms were restricted by suspending them in a 10 μL drop of M9 buffer. Isolated mitochondria were also illuminated in 10 uL drop at a concentration of 25 mg/mL. Identical acquisition conditions, including optics (2x objective), magnification (4x) and illumination settings on the X-Cite were used to standardize light intensity for all of the strains and conditions tested in this work. This intensity was measured empirically at 10.2 mW/mm^2^ using a calibrated silicon photodetector connected to an optical power meter (model number 818 P-010-12, Newport Corporation, Irvine, CA).

### Mitochondrial Isolation, enzymatic assays and respiration

Mitochondria were isolated from staged young adult *C. elegans*, as previously described using differential centrifugation in mannitol and sucrose-based media^48^,^49^.

Following isolation, mitochondrial respiration was measured using a Clark-type O_2_ electrode (Hansatech Instruments, UK) as previously described^48^. Briefly, mitochondria (1 mg/ml protein) were suspended in respiration buffer (120 mM KCl, 25 mM sucrose, 5 mM MgCl_2_, 5 mM KH_2_PO_4_, 1 mM EGTA, 10 mM HEPES, 1 mg/ml fat-free BSA, pH 7.3 at 25 °C). Complex-specific substrates (complex II, 5 mM succinate; complex IV, 2.5 mM/2.5 μM Ascorbate/TMPD) were used to initiate state 2 respiration. State 3 respiration was initiated with 200 μM ADP.

Isolated enzymatic activities of complex I, II and citrate synthase were determined spectrophotometrically using fresh mitochondria that were freeze-thawed as previously described. Complex I was calculated as the rotenone-sensitive rate of NADH-oxidation (ε = 6180 M^−1^ at 340 nm)^50^. Complex II activity was determined as the succinate-driven, malonate-sensitive rate of DCPIP-reduction (ε = 21000 M^−1^ at 600 nm)^18^. Citrate synthase activity was determined as the rate of DTNB-coenzyme A formation (ε = 13600 M^−1^ at 412 nm)^51^.

### Viability assays

To assess paraquat sensitivity, synchronized L1 larval worms were illuminated for the indicated time and placed on NGM plates seeded with OP50 and containing paraquat at 0–300 μM in 50 μM increments. Viability was scored after 4 days, with survival defined as the number of animals progressing beyond L4 in that period. For FCCP studies, synchronized L4 were illuminated for the indicated time and cultured on a NGM plate seeded with OP50 containing 1 μM FCCP. Viability was assessed 1 day later. A worm was defined as dead if it failed to move forward or backward in response to a gentle touch to the head.

### Brood size

Staged L4 worms were illuminated for the indicated amount of time and moved individually to fresh plate OP50-seeded NGM plates every 24 hours for 10 days. Progeny were counted on each plate 24–48 hours after removing the illuminated individual. For mating brood size assays, staged L4 worms were placed on a mating plate (containing a smaller amount of OP50, but food was not restrictive) with 10 transgenic males. The worms were moved to a new mating plate every 24-hours, and progeny were scored 24–48 hours after that. Cross-fertilized progeny were identified by the presence of GFP in the dopaminergic neurons.

## Additional Information

**How to cite this article**: Wojtovich, A. P. *et al*. Chromophore-Assisted Light Inactivation of Mitochondrial Electron Transport Chain Complex II in *Caenorhabditis elegans*. *Sci. Rep.* **6**, 29695; doi: 10.1038/srep29695 (2016).

## Supplementary Material

Supplementary Information
