# Supplementary material for: Chromophore-Assisted Light Inactivation of Mitochondrial Electron Transport Chain Complex II in Caenorhabditis elegans
Source: Sci Rep. 2016 Jul 21;6:29695. doi: 10.1038/srep29695 (PMC4954975; doi:10.1038/srep29695)
Supplement: Supplementary Information [file srep29695-s1.pdf]

Chromophore-Assisted Light Inactivation of Mitochondrial Electron Transport Chain Complex II in *Caenorhabditis elegans.*

Andrew P Wojtovich[1,2,*], Alicia Y Wei[1], Teresa A Sherman[3], Thomas H Foster[4], Keith Nehrke[2,3]

[1]University of Rochester Medical Center, Department of Anesthesiology, Rochester, 14642. United States of America.

[2]University of Rochester Medical Center, Department of Pharmacology and Physiology, Rochester, 14642, United States of America.

[3]University of Rochester Medical Center, Department of Medicine, Rochester, 14642, United States of America.

[4]University of Rochester Medical Center, Department of Imaging Sciences, Rochester, 14642, United States of America.

*Corresponding author

Andrew_Wojtovich@urmc.rochester.edu

Supplementary Figure S1.

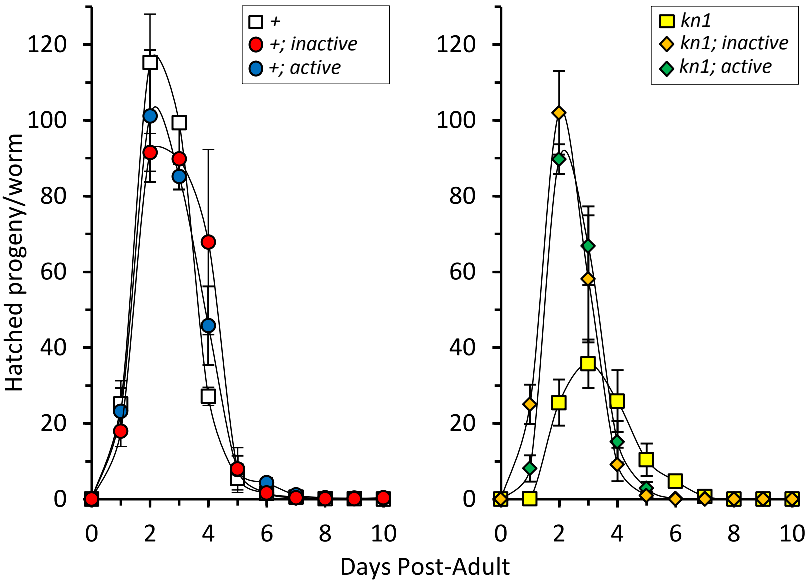

Supplementary Figure S2.

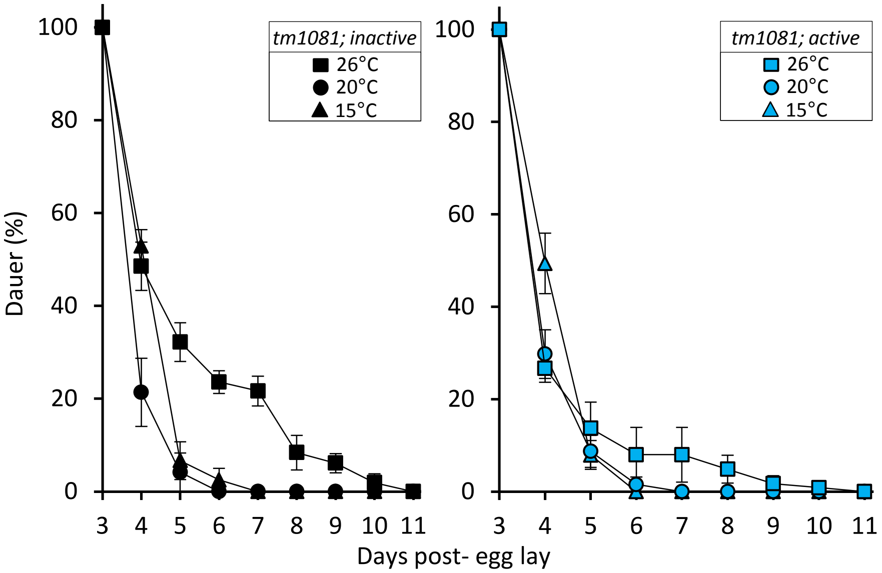

Supplementary Figure S3.

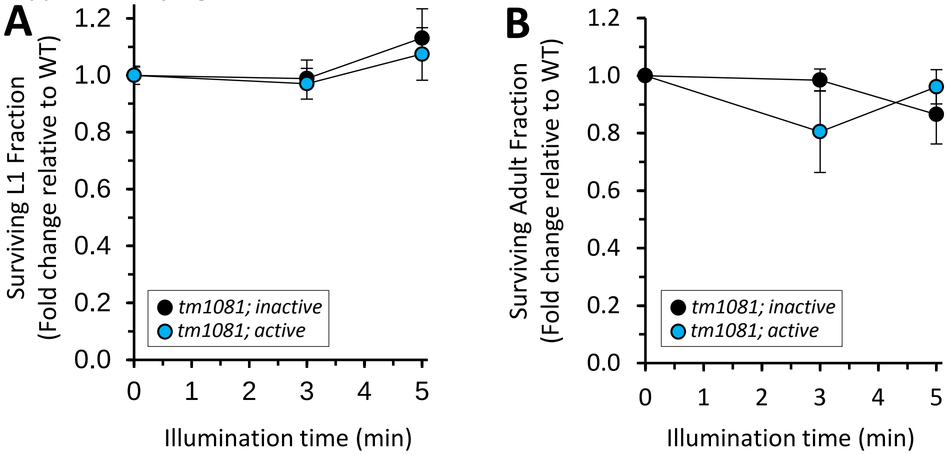

Supplementary Figure S4.

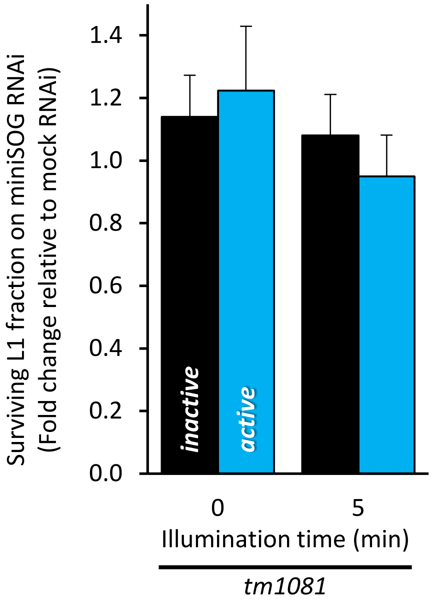

Supplementary Figure S5.

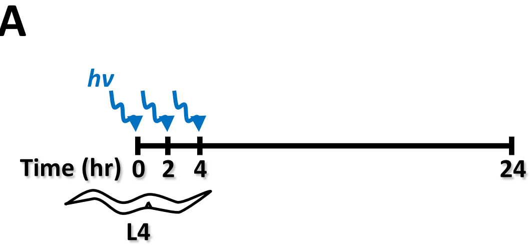

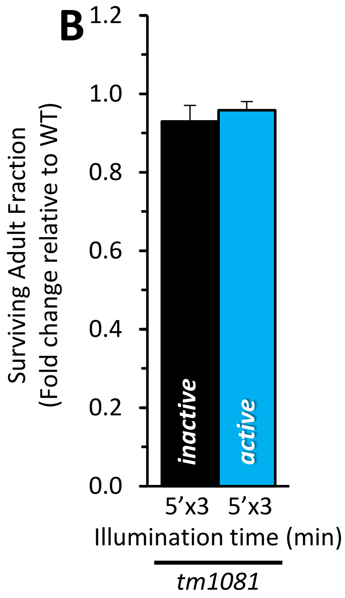

Supplementary Figure S6.

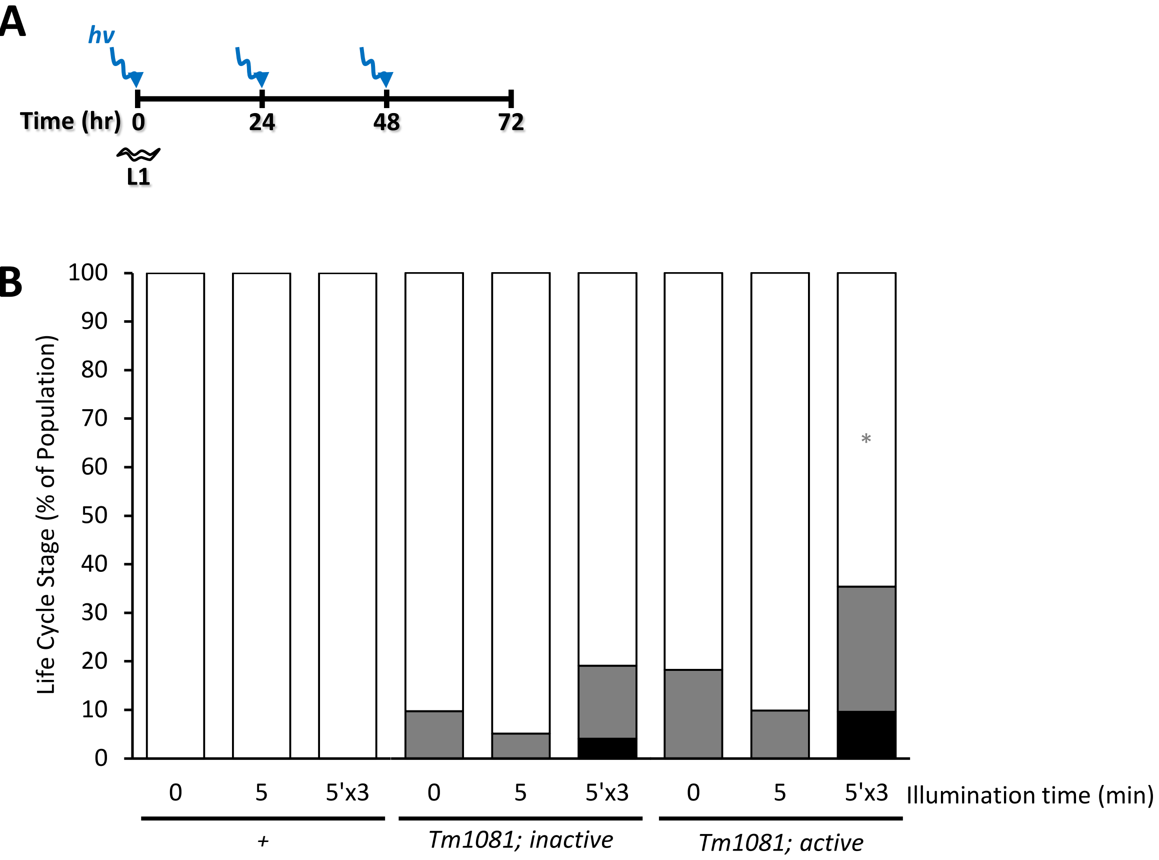

Supplementary Figure S7.

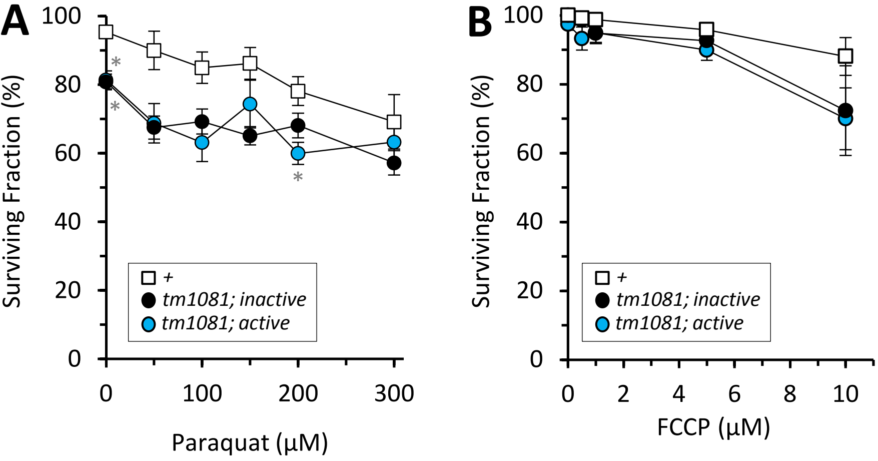

Supplementary Figure S8.

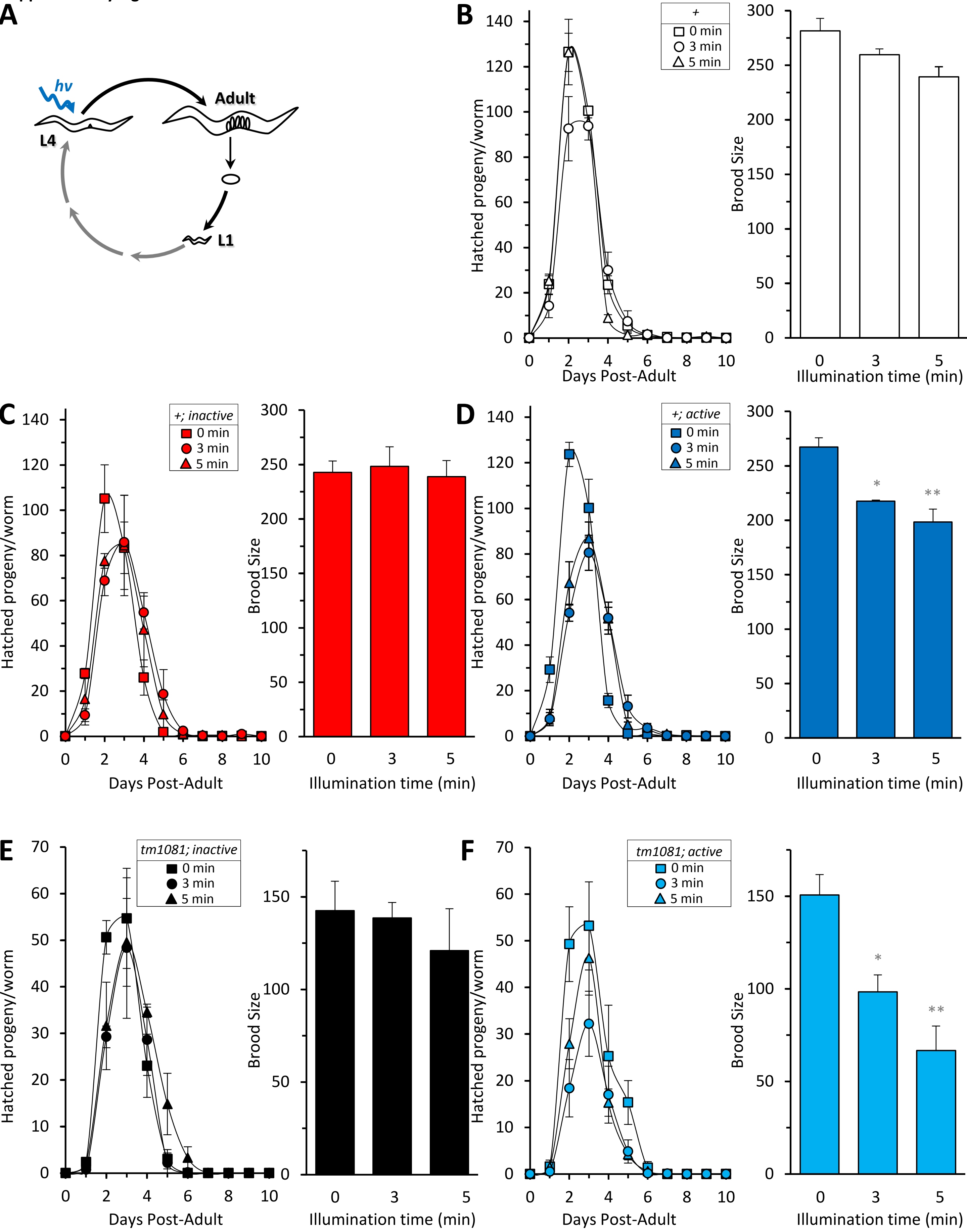

Strain	Genotype	Abbreviation	Notes
N2-Bristol	*mev-1(+)III*	*+*	Wild-type
TK22	*mev-1(kn1)III*	*kn1*	Paraquat-sensitive missense mutant allele [1]
KWN589	*mev-1(tm1081)III; hT2[bli-4(e937) let-?(q782) qIs48] (I;III)]*	*tm1081*	Lethal deletion allele, backcrossed 3x to N2 and balanced
KWN534	*rnySi19 [pTFA11(Pmev-1::mev-1::miniSOG(active), unc-119 (C. briggsae)) into ttTi5605 ChrII Mos site]*	*+; active*	Wild-type single-copy MosSCI *mev-1* transgene fused to active miniSOG, capable of CALI [2]
KWN465	*rnySi29 [pTFA12(Pmev-1::mev-1::miniSOG(inactive), unc-119 (C. briggsae)) into ttTi5605 ChrII Mos site]*	*+; inactive*	Wild-type single-copy MosSCI *mev-1* transgene fused to inactive miniSOG, not capable of CALI [2]
KWN510	*mev-1(kn1)III, rnySi19*	*kn1; active*	Reduced paraquat sensitivity compared to *kn1*
KWN512	*mev-1(kn1)III, rnySi29*	*kn1; inactive*	Reduced paraquat sensitivity compared to *kn1*
KWN530	*mev-1(tm1081)III, rnySi19*	*tm1081; active*	Viable
KWN519	*mev-1(tm1081)III, rnySi29*	*tm1081; inactive*	Viable
UR314	*him-5(e1490)V, egIs1[pdat-1:GFP]IV*		

Table S1. *C. elegans* strains and abbreviations. The wild-type *mev-1* gene (+) resides on chromosome III. The *mev-1(kn1)* and *mev-1(tm1081)* alleles contain a missense mutation or deletion, respectively. The *tm1081* allele was homozygous lethal and was maintained as a heterozygote using the hT2 chromosome I;III reciprocal translocation as a balancer. miniSOG was fused to the C-terminus of the wild-type *mev-1* gene and inserted on chromosome II using *Mos1*-mediated single copy insertion (MosSCI). Two variants (active and inactive) of miniSOG were used. The active variant is able to produce ROS in response to light while the inactive variant contains a Cys426 mutation and is not able to produce ROS. All strains were backcrossed to N2 at least 3 times prior to use.

1. Ishii N, Fujii M, Hartman PS, Tsuda M, Yasuda K, Senoo-Matsuda N, et al. A mutation in succinate dehydrogenase cytochrome b causes oxidative stress and ageing in nematodes. Nature. 1998;394(6694):694-7.
2. Shu X, Lev-Ram V, Deerinck TJ, Qi Y, Ramko EB, Davidson MW, et al. A genetically encoded tag for correlated light and electron microscopy of intact cells, tissues, and organisms. PLoS Biol. 2011;9(4):e1001041. doi: doi: 10.1371/journal.pbio.1001041.

Supplementary Figure Legends

Supplementary Figure S1: Daily brood size of *mev-1(kn1)III* complemented by *mev-1(+)::miniSOG* variants. Staged L4 worms were selected, and the number of viable progeny produced over the course of ten days was counted. Total brood size is presented in Figure 2B. Data are shown on separate axes for clarity. Data are mean±SEM. N= 4-7. An "N" is considered an independent trial of 1-5 worms. Alleles are abbreviated as outlined in Figure 1.

Supplementary Figure S2: Rescue of *mev-1(tm1081)* deletion strain with *mev-1(+)::miniSOG* results in a transient dauer phenotype. *Mev-1(+)::miniSOG* rescued the lethal *tm1081* deletion but also caused a modest incidence of transient temperature-sensitive dauer arrest. Following a synchronized 2-hour egg lay, dauer worms were selected 3 days later and moved to a fresh NGM plate seeded with OP50. Dauer worms were followed at indicated temperatures in the absence of light. Worms were removed when they exited dauer. Data are shown on separate axes for clarity. Data are mean±SEM. N=4. An "N" is considered an independent trial. Alleles are abbreviated as outlined in Figure 1.

Supplementary Figure S3: Acute CALI of complex II is not lethal to L1 or adult *C. elegans*. *C. elegans* expressing wild-type (+), *tm1081; inactive*, or *tm1081;active* variants of *mev-1* were illuminated, and viability was assessed. (A) Synchronized L1 worms were illuminated for the indicated time and cultured on a NGM plate seeded with OP50. Baseline survival in the absence of light: wild-type (+), 95±2% survival; *tm1081;inactive* , 81±2%; *tm1081;active*, 81±3%. Data are mean±SEM. N= 10-22. An "N" is considered an independent trial of >50 worms. Adjusted P>0.05 vs the no light or vs the light-matched inactive counterpart (2-way ANOVA with Sidak

multiple comparisons test). (B) Synchronized L4 were illuminated for the indicated time and cultured on a NGM plate seeded with OP50. Viability was assessed 1 day later. Baseline survival in the absence of light: wild-type (+), 100±0.0% survival; *tm1081;inactive*, 100±0.2%; *tm1081;inactive*, 97±1.2%. Data are mean±SEM. N= 7. An "N" is considered an independent trial of >50 worms. Adjusted P>0.05 vs the no light or vs the light-matched inactive counterpart (2-way ANOVA with Sidak multiple comparisons test). Alleles are abbreviated as outlined in Figure 1.

Supplementary Figure S4: Inhibition of MEV-1::miniSOG synthesis with RNAi targeting of miniSOG. Staged L1 worms expressing wild-type (+), *tm1081;inactive*, or *tm1081;active* variants of *mev-1* were illuminated for 0 or 5min. L1 were then cultured on plates containing either an empty vector or vector targeting miniSOG. Viability was assessed 72 hours later. Data are mean±SEM. N= 4. An "N" is considered an independent trial of >40 worms. There were no significant difference between groups - Adjusted P>0.05 vs no light or vs light-matched inactive counterpart (2-way ANOVA with Sidak multiple comparisons test). Alleles are abbreviated as outlined in Figure 1.

Supplementary Figure S5: Consecutive pulses are not lethal to adult *C. elegans*. *C. elegans* expressing wild-type (+), *tm1081;inactive*, or *tm1081;active* variants of *mev-1* were illuminated, and viability was assessed. (A) Experimental design. Synchronized L4 were illuminated for a 5 min period on three separate occasions as indicated and cultured on a NGM plate seeded with OP50. (B) Viability was assessed 1 day later. Baseline survival under consecutive pluses: wild-type (+), 97±0.9% survival; *tm1081;inactive*, 90±2.0%; *tm1081;active*, 90±3.9%. Data are mean±SEM. N= 3. An "N" is considered an independent trial of >50 worms.

Adjusted P>0.05 vs the no light (from Figure S3B) or vs a single 5 min exposure (from Figure S3B) or vs the light-matched inactive counterpart (2-way ANOVA with Sidak multiple comparisons test). Alleles are abbreviated as outlined in Figure 1.

Supplementary Figure S6: Consecutive pulses are not lethal to L1 *C. elegans*. *C. elegans* expressing wild-type (+), *tm1081;inactive*, or *tm1081;active* variants of *mev-1* were illuminated, and stage of the worm population was assessed. (A) Synchronized L1 worms were illuminated for 5 min every 24 hours as indicated and cultured on a NGM plate seeded with OP50. Following 3 days the stage of the worms was scored. As previously noted the *tm1081;inactive*, or *tm1081;active* strains have a propensity to enter dauer independent of light. Data are the mean of 4-9 independent trials of >40 worms Adjusted *P<0.05 vs the light-matched inactive counterpart (2-way ANOVA with Sidak multiple comparisons test). Alleles are abbreviated as outlined in Figure 1.

Supplementary Figure S7: Dose-response to paraquat and FCCP. (A) The sensitivity to paraquat was assessed in the absence of light. Staged L1 larva were cultured on plates containing various concentrations of paraquat, and survival was determined four days later. Data are mean±SEM. N= 4-22. An "N" is considered an independent trial of >50 worms. Wild-type (+) dose response is reproduced from Figure 2A for comparison purposes. * adjusted P<0.05 vs wild-type (+) (2-way ANOVA with Sidak multiple comparisons test). (B) Synchronized L4 were cultured on a NGM plate seeded with OP50 containing various concentrations of the ionophore FCCP. Survival was scored 1 day later. Data are mean±SEM. N=7. Adjusted P>0.05 vs wild-type (+) (2-way ANOVA with Sidak multiple comparisons test). 10 µM FCCP resulted in 28±16% survival of *mev-1(kn1)III* worms. Alleles are abbreviated as outlined in Figure 1.

Supplementary Figure S8: CALI of complex II decreases brood size. (A) *C. elegans* life cycle and schematic of light exposure. Staged L4 worms were illuminated (day 0) for the indicated time and placed onto a NGM plate seeded with OP50. Worms were moved daily to fresh plates over the course of 10 days. Each plate was scored for viable progeny the following day. Daily progeny count and total brood size are presented for (B) wild-type (+), (C) +*;inactive*, (D) +*;active*, (E) *tm1081;inactive*, (F) *tm1081;active*. Normalized data are presented in Figure 7. All data are mean±SEM. N= 4-6. An "N" is considered an independent trial of 1-5 worms. * adjusted $P<0.05$; ** adjusted $P<0.01$ vs no light (2-way ANOVA with Tukey multiple comparisons test). Alleles are abbreviated as outlined in Figure 1.

Supplementary Table S1: *C. elegans* strains and abbreviations. The wild-type *mev-1* gene (+) resides on chromosome III. The *mev-1(kn1)* and *mev-1(tm1081)* alleles contain a missense mutation or deletion, respectively. The *tm1081* allele was homozygous lethal and was maintained as a heterozygote using the hT2 chromosome I;III reciprocal translocation as a balancer. miniSOG was fused to the C-terminus of the wild-type *mev-1* gene and inserted on chromosome II using *Mos1*-mediated single copy insertion (MosSCI). Two variants (active and inactive) of miniSOG were used. The active variant is able to produce ROS in response to light while the inactive variant contains a Cys426 mutation and is not able to produce ROS. All strains were backcrossed to N2 at least 3 times prior to use.
